# Prognostic Factors and a Polymetastasis-Free Survival Nomogram in Oligometastatic Head and Neck Squamous Cell Carcinoma Treated with Local Ablative Therapy

**DOI:** 10.3390/curroncol33100585

**Published:** 2026-09-29

**Authors:** Young Seob Shin, Si Yeol Song, Seung Hoon Han, Minsu Kwon, Yoon Se Lee, Young Ho Jung, Seung-Ho Choi, Seyoung Seo, Sung-Bae Kim, Sang-wook Lee

**Affiliations:** 1 Department of Radiation Oncology, Asan Medical Center, University of Ulsan College of Medicine, Seoul 05505, Republic of Korea; speed686@amc.seoul.kr (Y.S.S.);; 2 Department of Otolaryngology-Head and Neck Surgery, Asan Medical Center, University of Ulsan College of Medicine, Seoul 05505, Republic of Korea; 3 Department of Oncology, Asan Medical Center, University of Ulsan College of Medicine, Seoul 05505, Republic of Korea

**Keywords:** oligometastasis, head and neck squamous cell carcinoma, local ablative therapy, polymetastatic conversion, nomogram

## Abstract

Evidence supporting metastasis-directed local ablative therapy (LAT) in head and neck squamous cell carcinoma (HNSCC) with oligometastatic disease (OMD) is limited. We retrospectively reviewed 93 patients with HNSCC who developed OMD and received LAT to all metastatic sites between 2006 and 2025. Clinical variables, sarcopenia, nutritional status, and inflammatory indices were evaluated as potential predictors of overall survival (OS), progression-free survival (PFS), polymetastasis-free survival (PMFS), and polymetastatic conversion (PC). An exploratory prognostic nomogram for PMFS was developed to inform treatment decision-making. After a median follow-up of 28.2 months, the 3-year OS and PMFS rates were 67.2% and 51.7%, respectively. Local failure occurred in 9 patients, while disease progression was predominantly distant. An HPV-positive oropharyngeal primary tumor was associated with improved OS and PMFS, whereas non-lung metastatic sites were adverse prognostic factors for PFS, and having two or more metastases was adverse for PC. Lower serum albumin levels correlated with worse OS and PFS, and an increasing systemic immune-inflammation index (SII) showed borderline significance for OS and PMFS. A PMFS nomogram based on four clinical parameters achieved an optimism-corrected concordance index of 0.66. Long-term survival and high local control were observed in this selected cohort of HNSCC patients with OMD treated with LAT. Tumor type, metastatic site, tumor burden, and host nutritional and inflammatory indices were associated with survival and progression to PC.

## Simple Summary

The benefit of metastasis-directed local ablative therapy in oligometastatic disease has been increasingly recognized, but evidence in head and neck squamous cell carcinoma remains limited. In this retrospective study of 93 selected patients treated with local ablative therapy, we observed favorable local control and long-term survival. Clinicopathologic factors including human papillomavirus-positive oropharyngeal cancer and lung-confined metastasis, together with nutritional status and blood markers of inflammation, were identified as prognostic factors. We propose an exploratory scoring chart based on four routinely available parameters that estimates how long a patient is likely to remain free of extensive metastatic spread, which may inform clinical decision-making.

## 1. Introduction

Oligometastatic disease (OMD) represents an intermediate state between localized and widely metastatic disease, characterized by a limited number and site of metastases. The prevailing paradigm suggests that metastasis-directed ablative local therapy can alter the natural history of the disease and prolong survival. Randomized evidence in various solid tumors, such as the SABR-COMET trial, has supported this benefit [1]. However, the effectiveness of this approach has been inconsistent across different tumor types and treatment settings [2,3,4], and the degree of benefit varies among patients. Therefore, appropriate patient selection is crucial.

Head and neck cancer has remained relatively understudied in this context. Distant metastasis occurs in fewer than one in ten patients during the course of the disease, usually metachronously, and only about half of these cases are oligometastatic [5]. Moreover, the disease is heterogeneous, arising from diverse anatomical subsites and following distinct clinical courses according to viral association. The available evidence is largely retrospective and, although generally reporting favorable outcomes, offers limited guidance on how to optimally deploy the available treatment options—local ablative therapy (LAT), systemic therapy, or their combination—and for which patients.

Beyond tumor characteristics, the patient’s overall physiological condition may inform treatment decisions. Performance status is consistently prognostic but is subjective and semi-quantitative; objective, quantitative surrogates of host condition—such as CT-defined sarcopenia and nutritional or inflammatory indices—can be complementary or even superior to performance status. These markers are established prognostic factors in head and neck cancer and, more broadly, in patients with metastatic disease across diverse primary cancers, including in metastasis-directed treatment settings [6,7,8]. However, whether they improve patient selection in head and neck OMD has been scarcely examined.

We therefore conducted a retrospective study of patients with head and neck squamous cell carcinoma (HNSCC) who developed OMD and underwent LAT to all metastatic sites. We aimed to describe their oncological outcomes and identify factors associated with disease progression and prognosis, including tumor burden, primary tumor subtype, and objective host condition markers (e.g., skeletal muscle index and nutritional/inflammatory indices).

## 2. Materials and Methods

### 2.1. Patients

We retrospectively reviewed patients with HNSCC who developed OMD and underwent LAT to all metastatic lesions at Asan Medical Center between January 2006 and September 2025. Eligible patients met the following criteria: (i) pathologically confirmed HNSCC; (ii) genuine, de novo OMD according to the ESTRO–EORTC consensus classification, with no prior history of polymetastatic or oligometastatic disease [9], defined as up to five metastatic lesions in no more than three organs; and (iii) LAT delivered to all metastatic sites with the intent of achieving durable local control, including stereotactic body radiotherapy (SBRT), surgical metastasectomy, radiofrequency ablation (RFA), or conventional radiotherapy. Synchronous and metachronous OMD were distinguished using a 6-month interval from the primary cancer diagnosis. Patients were excluded if they had non-squamous histology, less than 3 months of follow-up after local therapy, or incomplete local therapy (i.e., not all metastatic lesions were treated). A total of 93 patients met these criteria and constituted the study cohort. The study was approved by the Institutional Review Board of Asan Medical Center (approval no. 20251097), with a waiver of informed consent due to the retrospective design.

### 2.2. Treatment and Evaluation

The application of LAT, the choice of modality, and its combination with systemic therapy were not protocol-defined but were determined by the treating physician based on the extent of disease and the patient’s medical condition. The local treatment modalities included surgical metastasectomy, SBRT, conventional radiotherapy, and RFA. Conventional radiotherapy was included when treatment was directed to all metastatic sites with the intent of achieving durable local control and its biologically effective dose (BED10) exceeded that of a commonly prescribed palliative regimen (30 Gy in 10 fractions). BED10 was calculated as BED10 = D × (1 + d/[α/β]), where α/β = 10 Gy, D is the total dose, and d is the dose per fraction. Following treatment, all patients were regularly followed at 3- to 6-month intervals for up to 5 years. Evaluations included physical examination, complete blood count, standard blood chemistry panel, and cross-sectional imaging (CT and/or MRI) of the primary and metastatic sites, with additional assessments as clinically indicated. Treatment-related adverse events were graded according to the Common Terminology Criteria for Adverse Events (CTCAE) version 5.0. The last follow-up date was defined as the date of the last clinical or imaging assessment. The vital status of surviving patients was confirmed through the national health database, with living patients censored at the date of last vital-status confirmation (1 May 2026).

### 2.3. Body Composition and Inflammatory Indices

Skeletal muscle was quantified on a single axial chest CT slice at the twelfth thoracic vertebra (T12), selected by the investigator at the level where the twelfth rib attaches to the vertebral body, both at diagnosis and at OMD diagnosis. Segmentation was performed using a publicly available deep-learning model for CT muscle and fat segmentation [10], which we adapted for single-slice T12 assessment with additional post-processing. Each segmentation was visually reviewed by the investigator using a color overlay, and cases with organ leakage were manually corrected. The skeletal muscle index was calculated as the muscle cross-sectional area divided by height squared (cm^2^/m^2^). The neutrophil-to-lymphocyte ratio (NLR), the prognostic nutritional index (PNI = 10 × serum albumin [g/dL] + 0.005 × total lymphocyte count [/µL]), and the systemic immune-inflammation index (SII = platelet count × neutrophil count ÷ lymphocyte count) were computed from the corresponding laboratory values. For all body composition and inflammatory measures, the rate of change per month between the two time points was derived.

### 2.4. Statistical Analysis

The primary endpoint was overall survival (OS), and secondary endpoints included polymetastatic conversion (PC), polymetastasis-free survival (PMFS), progression-free survival (PFS), and local control (LC), all measured from the time of OMD diagnosis. PC was defined as progression beyond the oligometastatic limits (i.e., more than five lesions or involvement of more than three organs). PMFS was defined as the time to polymetastatic conversion or death from any cause, whichever occurred first. LC was defined as freedom from failure within a treated lesion. For PC and LC, death prior to the event was treated as a competing risk.

Continuous variables are summarized as median [interquartile range (IQR)] or mean ± standard deviation, and categorical variables as counts (percentages). Oropharyngeal carcinomas with unknown p16/HPV status were included in the other HNSCC group for prognostic analyses and in the No category of the nomogram. For the competing-risk endpoints (PC and LC), cumulative incidence functions were estimated and compared using Gray’s test, and Fine-Gray subdistribution hazard models were fitted with death as the competing event. OS, PFS, and PMFS were analyzed using the Kaplan–Meier method with the log-rank test and Cox proportional hazards regression. Variables with a univariate *p*-value < 0.10 were included in multivariable models, from which the final models were derived by Akaike information criterion (AIC)-based backward elimination. Treatment-related variables were excluded from candidate prognostic factors to focus on patient- and disease-related predictors among patients receiving LAT, as treatment selection was individualized according to patient and disease characteristics. Rate-of-change variables were expressed per one standard deviation rather than per native unit. A prognostic model for PMFS was constructed using multivariable Cox regression and presented as a nomogram (rms package); it was internally validated by bootstrap resampling (1000 replicates) to obtain an optimism-corrected concordance index and a calibration plot. Candidate predictors for the nomogram were variables associated with PMFS or polymetastatic conversion in the univariable analyses (HPV-positive oropharyngeal primary tumor, metastatic site, number of metastases, and largest metastatic diameter). Nested models were compared using the optimism-corrected concordance index, and the model with the highest corrected discrimination was selected; adding further variables did not improve corrected discrimination.

All analyses were performed using R software, version 4.2.2 (R Foundation, Vienna, Austria; http://www.R-project.org/, accessed on 27 September 2026). Two-sided *p*-values less than 0.05 were considered statistically significant.

## 3. Results

### 3.1. Patient Characteristics

A total of 93 patients were included (Table 1). The median age was 59 years (IQR, 55–66) at initial diagnosis and 62 years (IQR, 57–68) at LAT, and 76 patients (81.7%) were male. Most had metachronous disease (*n* = 81, 87.1%), with a median disease-free interval of 21.3 months (IQR, 13.1–34.0) from initial diagnosis to OMD diagnosis. The most common primary sites, in descending order, were the nasopharynx (*n* = 27, 29.0%), oropharynx (*n* = 21, 22.6%; 11 HPV-positive), larynx (*n* = 15, 16.1%), and oral cavity (*n* = 14, 15.1%). For analysis, tumors were grouped as nasopharyngeal carcinoma (*n* = 27, 29.0%), HPV-positive oropharyngeal carcinoma (*n* = 11, 11.8%), and other HNSCC (*n* = 55, 59.1%).

At the time of OMD diagnosis, most patients had a single metastatic lesion (*n* = 77, 82.8%; ≥2 lesions in 16 patients, 17.2%) confined to a single organ (*n* = 91, 97.8%). The most frequently involved metastatic sites were the lung only (*n* = 65, 69.9%), bone (*n* = 12, 12.9%), liver (*n* = 6, 6.5%), and distant lymph nodes (*n* = 5, 5.4%). The median diameter of the largest metastasis was 1.6 cm (IQR, 1.2–2.5 cm). LAT included SBRT in 41 patients (44.1%), surgical metastasectomy in 34 patients (36.6%), conventional radiotherapy in 13 patients (14.0%), and radiofrequency ablation in 5 patients (5.4%). The dose/fractionation schedules used for SBRT and conventional radiotherapy are summarized in Supplementary Table S1. Systemic therapy was administered before or concurrently with local therapy in a subset of patients (before, *n* = 20 [21.5%]; concurrent, *n* = 9 [9.7%]) and after local therapy in 7 patients (7.5%). Overall, 28 patients (30.1%) received systemic therapy at some point, whereas 65 patients (69.9%) received LAT alone.

Body composition, nutritional, and inflammatory markers are summarized in Supplementary Table S2. The skeletal muscle index, as well as the nutritional and inflammatory indices, were available for 89–93 patients at OMD diagnosis and for 86–87 patients at initial diagnosis. Rate-of-change values for these indices were estimable in 71–75 of the 81 metachronous patients.

### 3.2. Survival and Polymetastatic Conversion

**Figure 1:**
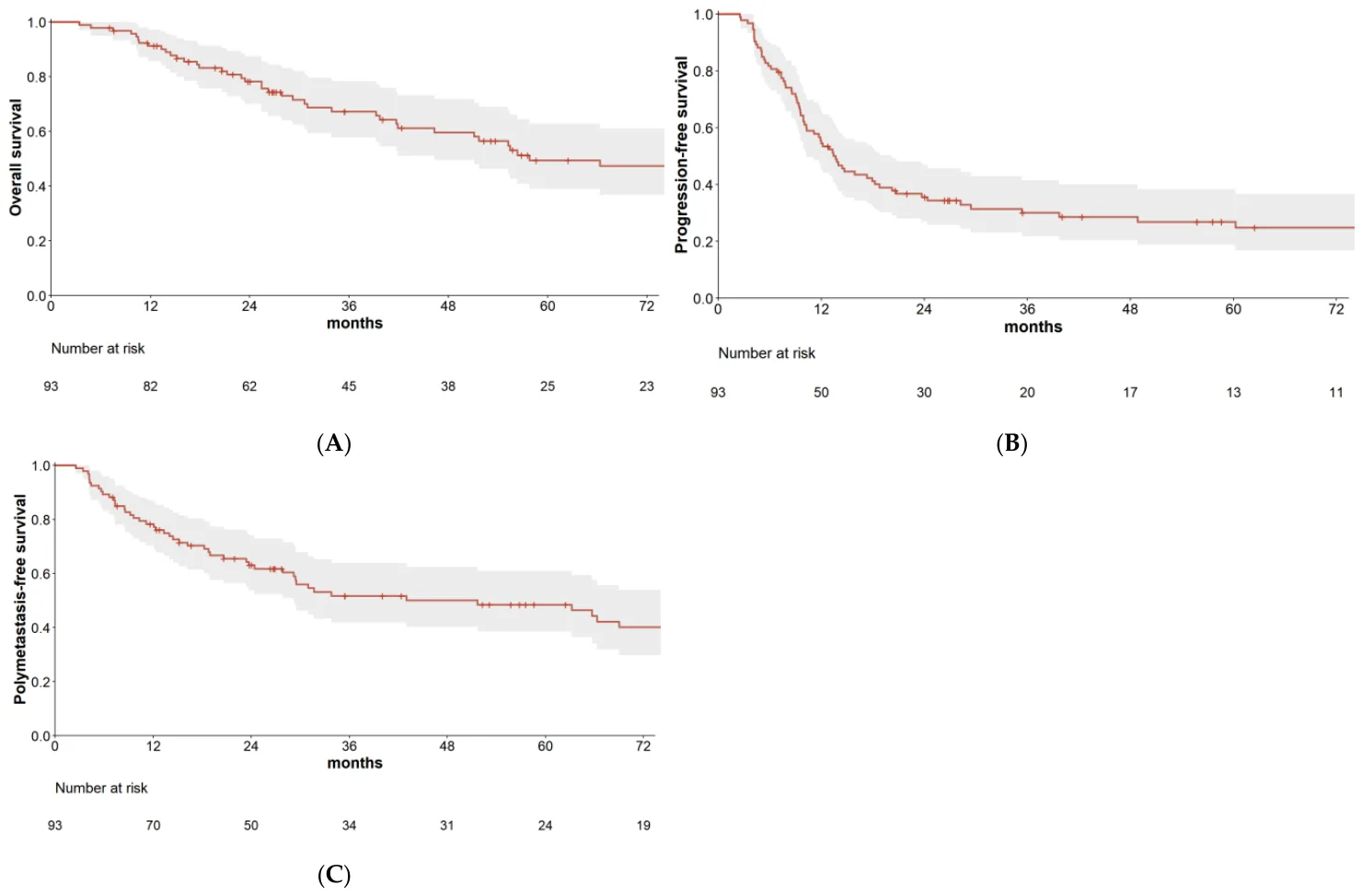
Kaplan–Meier curves depicting overall survival (**A**), progression-free survival (**B**), and polymetastasis-free survival (**C**) in the entire cohort (N = 93). Shaded areas represent 95% confidence intervals.

**Figure 2:**
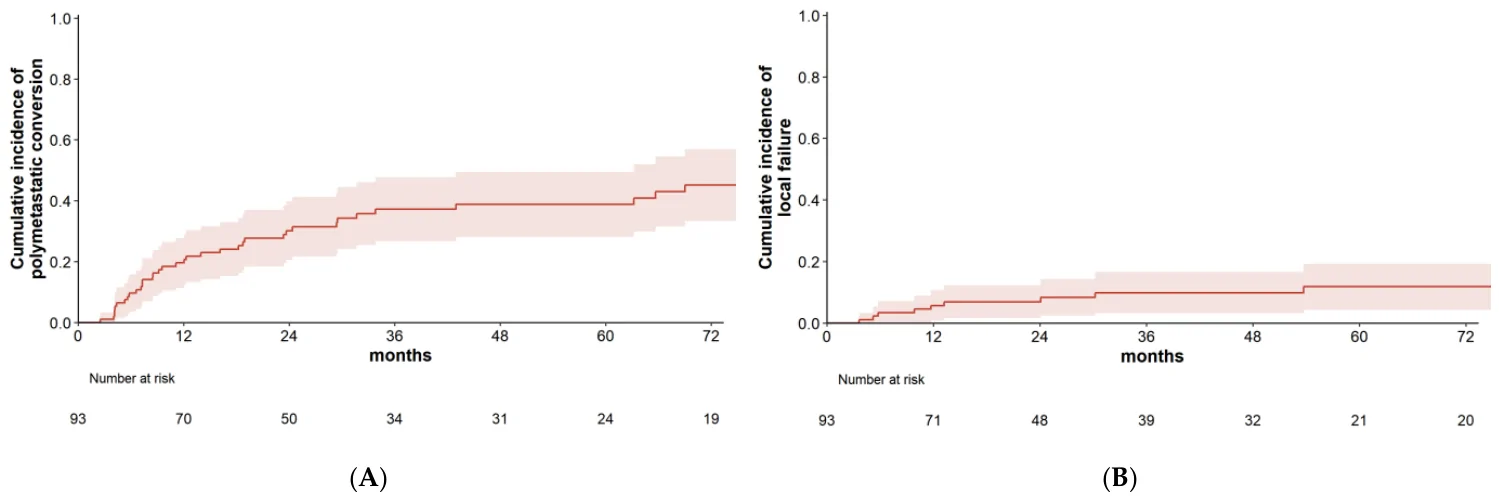
Cumulative incidence of polymetastatic conversion (**A**) and local failure (**B**) for the entire cohort (N = 93), with death treated as a competing event. Shaded areas represent 95% confidence intervals.

After a median follow-up of 28.2 months (range, 2.3–203.6), 51 patients (54.8%) had died. The median OS was 57.8 months, with 1- and 3-year OS rates of 91.3% and 67.2%, respectively. The median PFS was 13.6 months (1-year, 54.5%; 3-year, 30.0%), and the median PMFS was 51.7 months (1-year, 78.3%; 3-year, 51.7%) (Figure 1). PC occurred in 39 patients; with death treated as a competing event, the cumulative incidence of conversion was 19.5% at 1 year and 37.2% at 3 years (Figure 2A). Survival differed by primary tumor group, with the most favorable outcomes in patients with an HPV-positive oropharyngeal primary, followed by nasopharyngeal and other primaries (Supplementary Figure S1).

### 3.3. Patterns of Failure

Disease progression of any type occurred in 64 patients (68.8%; Table 2). The first site of failure was distant metastasis alone in 51 patients, isolated head and neck recurrence in 5, isolated local failure in 4, and combined sites in 4. Overall, local failure within a treated lesion occurred in 9 patients, head and neck recurrence in 8, and distant metastasis in 57. Distant metastasis was by far the most common first site of failure. Among the 57 patients who developed distant metastasis, the first distant site involved the same organ affected at OMD diagnosis in 28 (49.1%), a different organ in 14 (24.6%), and both in 15 (26.3%). Twenty patients underwent repeat LAT for all recurrent sites of oligometastatic recurrence; 12 regained a disease-free state and remained so until the last follow-up, whereas 8 subsequently developed polymetastatic disease. Seven patients received planned systemic therapy after local treatment. Another 44 began systemic therapy for progressive disease at a median of 10.3 months (IQR, 6.7–23.0) from OMD diagnosis.

### 3.4. Local Control and Toxicity

Local failure occurred in 9 of 93 patients. The 2-year and crude cumulative incidences of local failure were 8.2% and 9.7%, respectively (Figure 2B). By treatment modality, crude LC was highest with SBRT (97.6%; 1 of 41 patients), followed by surgical metastasectomy (88.2%; 4/34), RFA (80.0%; 1/5), and conventional radiotherapy (76.9%; 3/13). Over the study period, the proportion of patients treated with SBRT increased from 28.1% before 2016 to 64.7% during 2021–2025, while the use of surgery and RFA declined. This shift corresponded with a decrease in the size of treated metastases (median largest diameter: 2.2 cm before 2016 vs. 1.3 cm in 2021–2025; *p* = 0.001). The only potential high-grade event was the death of one patient from pneumonia 1.5 months after pulmonary metastasectomy, although its relationship to treatment was unclear. No other grade ≥3 toxicity attributable to metastasis-directed local therapy was observed.

### 3.5. Prognostic Factors

In the entire cohort, an HPV-positive oropharyngeal primary tumor was the only factor significantly associated with OS (hazard ratio [HR] 0.23, 95% confidence interval [CI] 0.05–0.95; Table 3, Supplementary Table S3). For PFS, synchronous presentation, non-lung metastatic site, and lower albumin and hemoglobin levels at OMD diagnosis were associated on univariate analysis. On multivariable analysis, non-lung site (HR 1.78, 95% CI 1.06–2.99) and albumin (HR 0.42, 95% CI 0.18–0.98) remained significant. Regarding PMFS, HPV-positive oropharynx, larger metastatic size, and non-lung site were associated on univariate analysis; however, only HPV-positive oropharynx remained significant on multivariable analysis (HR 0.22, 95% CI 0.05–0.93), while non-lung site showed a trend toward significance (HR 1.88, 95% CI 1.00–3.55). In Fine-Gray competing-risk analysis, ≥2 metastases (subdistribution hazard ratio [sHR] 2.51, 95% CI 1.22–5.16) and a non-lung site (sHR 2.25, 95% CI 1.09–4.65) were associated with polymetastatic conversion (Supplementary Table S4).

In the metachronous subset (Table 4, Supplementary Table S5), HPV-positive oropharynx, lower albumin at OMD diagnosis, and the NLR and SII rates were associated with OS in univariate analysis. HPV-positive oropharynx (HR 0.23, 95% CI 0.05–0.96) and lower albumin (HR 0.36, 95% CI 0.14–0.96) remained significant in multivariable analysis, while the SII rate showed borderline significance (HR 1.32, 95% CI 0.96–1.81). For PFS, lower albumin, hemoglobin, and PNI at OMD diagnosis, as well as the NLR rate, were associated in univariate analysis; however, albumin was the only factor retained in multivariable analysis (HR 0.25, 95% CI 0.10–0.64). Regarding PMFS, HPV-positive oropharynx, larger tumor size, non-lung metastatic site, and the NLR and SII rates were associated in univariate analysis. In multivariable analysis, HPV-positive oropharynx (HR 0.26, 95% CI 0.06–1.12) and the SII rate (HR 1.33, 95% CI 0.98–1.81) showed a trend toward significance. Fine-Gray competing-risk analysis revealed that ≥2 metastases (sHR 3.59, 95% CI 1.72–7.49), larger metastatic size (sHR 1.32, 95% CI 1.10–1.58), and longer disease-free interval (per month; sHR 1.01, 95% CI 1.00–1.02) were associated with polymetastatic conversion (Supplementary Table S4).

### 3.6. Prognostic Nomogram

**Figure 3:**
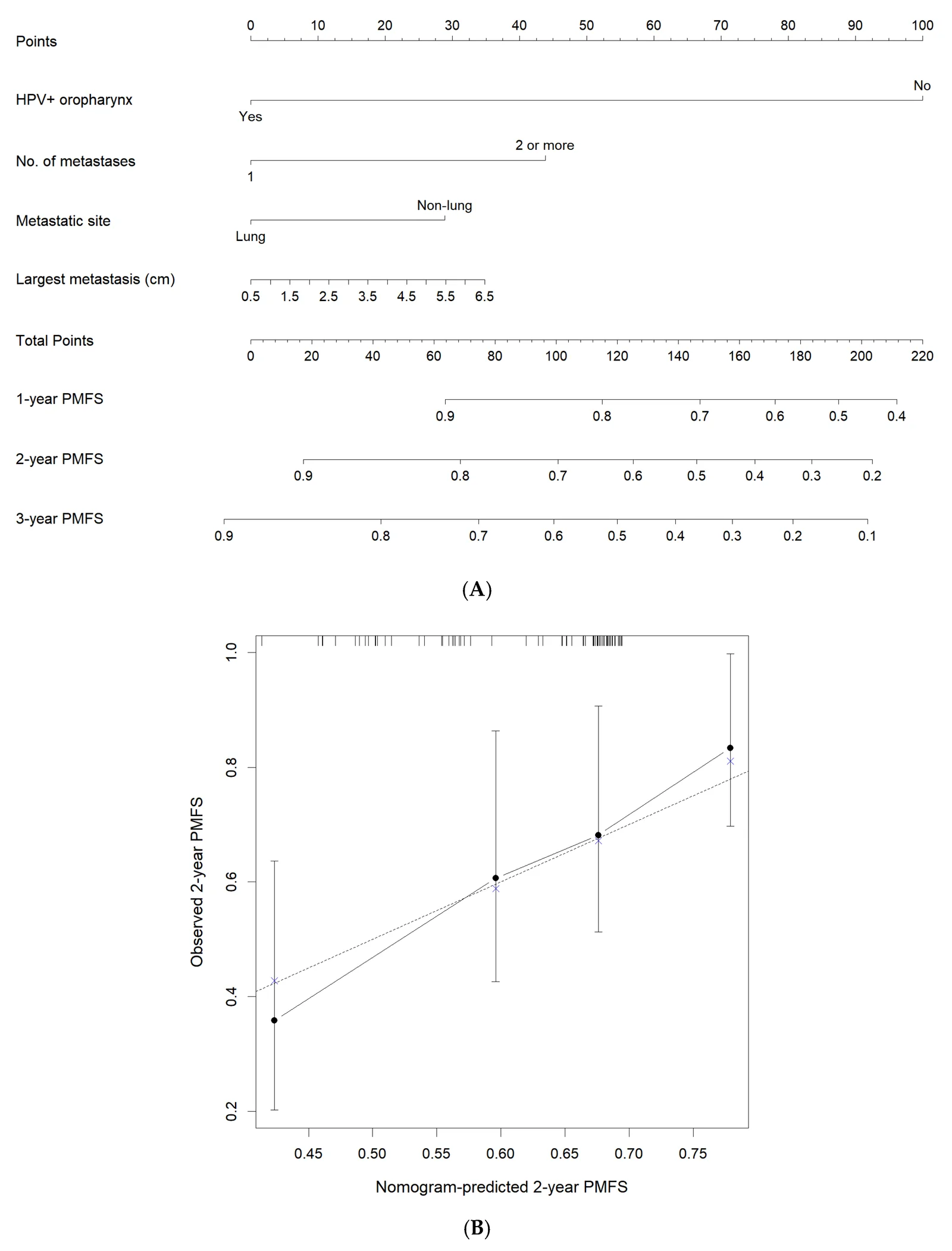
Nomogram for predicting 1-, 2-, and 3-year polymetastasis-free survival (PMFS) based on human papillomavirus (HPV)-positive oropharyngeal primary tumor status, number of metastases, metastatic site, and largest metastatic diameter (**A**). The calibration plot compares predicted and observed 2-year PMFS (**B**).

A prognostic nomogram for PMFS was developed using the entire cohort, incorporating HPV-positive oropharyngeal primary tumors, non-lung metastatic site, ≥2 metastases, and the largest metastatic lesion size to estimate 1-, 2-, and 3-year PMFS (Figure 3A). Nasopharyngeal carcinoma was evaluated as a distinct primary-tumor category in the prognostic analyses, but including it as a separate category did not improve the predictive performance of the nomogram. The model was fitted in all 93 patients with 56 PMFS events. The optimism-corrected concordance index was 0.66 based on 1000 bootstrap replications, demonstrating moderate agreement between predicted and observed 2-year PMFS on the calibration plot (Figure 3B). In the metachronous subset, where dynamic rate-of-change indices could be evaluated, adding these indices did not improve discrimination compared to the model containing the same variables as the whole-cohort nomogram.

## 4. Discussion

LAT has garnered increasing interest in the management of OMD; however, OMD is a heterogeneous condition, and tools to guide individualized treatment remain limited. In our selected cohort of 93 patients with HNSCC treated with LAT to all metastatic sites, long-term survival and high local control were observed, although disease progression remained common and was predominantly distant. Prognosis was associated with disease characteristics and patient condition: an HPV-positive oropharyngeal primary tumor was favorable, whereas non-lung metastatic sites and a higher metastatic burden (≥2 lesions, larger size) were adverse factors. Additionally, laboratory-derived nutritional and inflammatory indices, such as serum albumin and the systemic immune-inflammation index, were also associated with outcomes—both at a single time point and in terms of their rate of change. We constructed a nomogram to estimate PMFS among patients treated with LAT, based on HPV-positive oropharynx, non-lung metastatic site, ≥2 metastases, and largest metastatic lesion size.

OS in our cohort was favorable: the median OS of 57.8 months is at the favorable end of published head and neck cancer series, where median OS typically ranges from approximately 23 to 47 months in squamous cell carcinoma and is higher in nasopharyngeal and HPV-associated diseases [11,12,13,14]. However, given the predominance of solitary, small, and lung-confined metastases in our cohort, this favorable survival could reflect case-mix as much as the effect of LAT itself.

We adopted PMFS as an endpoint based on the premise that the benefit of LAT is greatest in patients who remain in the oligometastatic state for a prolonged period. In this context, our outcomes are notable: although 69.9% of patients received LAT without systemic therapy, the 3-year PFS and PMFS reached 30.0% and 51.7%, respectively, with both curves plateauing thereafter. This indicates that a substantial proportion of patients in this selected cohort did not experience rapid systemic progression. These findings are consistent with those of OMET, the only randomized trial in head and neck oligometastasis, in which stereotactic ablative radiotherapy without chemotherapy achieved OS comparable to chemoradiotherapy (42 versus 41 months) but with substantially lower toxicity and cost [15].

In our cohort, local control was high across treatment modalities, with local failure occurring in only 9 of 93 patients. Although local control appeared highest following SBRT, the increased use of SBRT over time coincided with a decrease in the size of treated metastases. Therefore, this difference likely reflects confounding factors related to treatment era and lesion size rather than the true superiority of any technique. Disease progression was predominantly distant, including recurrences in organs already affected at the time of oligometastatic diagnosis. This pattern is consistent with previous studies, which reported in-field local failure rates below 15%, distant recurrence rates exceeding 50%, and a lung-predominant distribution [12,16]. In a substantial proportion of patients, failure occurred at still-limited sites amenable to local therapy. Twenty of the 64 patients with disease progression underwent repeat LAT for all recurrent sites of oligometastatic recurrence, and 12 of them remained disease-free until the last follow-up, whereas 8 subsequently developed polymetastatic disease. This proportion is consistent with other reports showing 27–37% repeat-ablation rates and occasional long-term cures [14,17]. These findings indicate that durable disease control can be observed in selected patients following local therapy, including repeat LAT, and suggest that oligometastatic recurrence after LAT may warrant consideration of repeat local therapy rather than being regarded as an indication for systemic therapy alone.

Although a significant proportion of patients with head and neck OMD benefit from LAT, the extent of this benefit varies among individuals, and the patients who derive the greatest advantage remain incompletely characterized. In our cohort, the most favorable prognostic factors were an HPV-positive oropharyngeal primary tumor and lung-limited metastatic disease. HPV/p16 positivity is the most consistently reported favorable factor in this setting [18,19], while non-pulmonary metastases—particularly those involving the liver, bone, or brain—have been associated with poorer outcomes compared with lung-confined disease [20]. The number of metastases is a key criterion for offering LAT; however, its role in further stratifying prognosis within the oligometastatic range of up to five lesions remains controversial. Some studies report that a higher number of metastases is prognostic [21], whereas others find that the number of involved organs provides more informative prognostic value [12,22]. In our cohort, the number of metastases predicted PC but was not associated with overall survival. Nevertheless, it improved the discriminatory power of our nomogram, suggesting it retains prognostic significance. However, the small number of patients with multiple lesions or multi-organ involvement limits definitive conclusions, and further study in larger cohorts is warranted.

A central challenge in OMD is identifying, before initiating LAT, the patients whose disease will disseminate rapidly or who will poorly tolerate treatment. Performance status has been reported as a host-related prognostic factor in many studies; however, it is subjective and may not provide sufficient discrimination among patients. We therefore examined whether body composition and laboratory-derived nutritional and inflammatory indices could more objectively capture the underlying physiological state. Systemic inflammation fosters a tumor microenvironment that promotes cancer progression and reflects diminished immune surveillance, both of which are indicated by the relative proportions of circulating blood cells [23]. Conversely, a declining nutritional reserve and loss of skeletal muscle signify a cachectic state characterized by reduced physiological reserve, leaving patients vulnerable to treatment intolerance and functional decline [6,8].

In our cohort, serum albumin at the time of OMD diagnosis was the most consistent host prognostic factor, independently associated with PFS. We also examined the rate of change in these indices, hypothesizing that a deteriorating host condition might become apparent before overt rapid disease progression. The SII rate showed borderline significance for both OS and PMFS, and several other single-time-point markers and rate indices were significant in univariate analysis. However, in multivariable analysis, only one marker was retained, likely reflecting the strong correlation among these variables. To date, we have identified only one study reporting the neutrophil-to-lymphocyte ratio as a prognostic factor in head and neck OMD [24]. Although these indices did not reach firm statistical significance, the moderate size of our cohort may have limited the power to confirm their independent prognostic value, and they warrant further investigation in larger studies.

Building on the prognostic factors identified above, we developed an exploratory prognostic model to estimate PMFS, which may inform clinical decision-making. Since the benefit of LAT is likely greatest in patients whose progression to polymetastatic disease is delayed—allowing them to survive longer without becoming candidates for systemic therapy—we considered PMFS the most appropriate endpoint. The nomogram was based on an HPV-positive oropharyngeal primary tumor, a non-lung metastatic site, the presence of two or more metastases, and the size of the largest metastasis. Although the SII was a borderline predictor of PMFS, it did not improve the model’s discriminatory power. This is because it provides little differentiation among the majority of patients whose values are near the median; however, in the minority whose SII trajectory deviates significantly from the median, it may still meaningfully inform clinical decisions. Previous attempts to build prognostic models in head and neck OMD have been limited to nasopharyngeal carcinoma and have predicted OS or PFS using parameters such as EBV DNA, LDH, age, and disease burden [25,26]. In contrast, our model was developed across HNSCC of various primary subsites using variables readily obtained in routine clinical care, which may enhance its generalizability. Nonetheless, given its moderate discriminatory ability and derivation from a single, modestly sized cohort, the nomogram should be regarded as an exploratory prognostic model that requires external validation before it can guide treatment selection.

This study has several limitations. First, it was retrospective, and the choice of treatment was inevitably influenced by each patient’s condition. Treatment modality and previous systemic therapy were excluded from prognostic modeling to focus on patient- and disease-related factors, but residual confounding by treatment selection cannot be excluded. Conventional radiotherapy may have provided less durable local control than other LAT modalities, potentially reflecting lesion characteristics, patient suitability, and evolving use of stereotactic techniques. Because only patients who underwent LAT were included, our findings are informative for this selected group but may not represent the entire population of patients with head and neck OMD. The predominance of small, solitary, and lung-confined metastases may reflect selection of patients with a lower metastatic burden and potentially more indolent disease. Without a non-LAT comparator group, the contribution of LAT to the favorable survival observed cannot be distinguished from the effects of patient selection. Accordingly, the nomogram predicts outcome conditional on having received LAT, not the incremental benefit of LAT over systemic therapy alone; quantifying such benefit would require randomized allocation or a matched comparison with patients managed without LAT. Second, because patients were accrued over an extended period, they were treated across an evolving therapeutic landscape, including advances such as the addition of immunotherapy. In an exploratory analysis, more recent treatment eras were associated with better overall and polymetastasis-free survival on univariable analysis, but this association did not persist in multivariable models. We acknowledge that some degree of era-related confounding is likely; however, it may be partly reflected in other covariates, as metastatic tumor size decreased significantly in more recently diagnosed patients. Third, the modest sample size and limited numbers of events reduced the statistical power and precision of the multivariable analyses. Associations near the significance threshold should therefore be interpreted cautiously, considering their effect estimates and confidence intervals, while nonsignificant findings do not exclude potentially meaningful associations. Confirmation in larger cohorts is needed. In addition, although developing the model across the full spectrum of HNSCC lends it broad applicability, it may not fully capture the distinct behavior of each disease entity, particularly given the small subgroups defined by HPV status and primary tumor site. In particular, the nomogram grouped oropharyngeal carcinomas with unknown HPV status and nasopharyngeal carcinomas with other HNSCC, potentially limiting subgroup-specific prognostic accuracy. Finally, T12 was selected because L3 imaging was frequently unavailable and C3 measurements could be affected by local disease and treatment-related changes. T12-based muscle assessment and sarcopenia cutoffs are less extensively validated in HNSCC, which may affect sarcopenia classification and comparability with previous studies.

## 5. Conclusions

In this selected cohort of patients with HNSCC and OMD treated with LAT to all metastatic sites, long-term survival and high local control were observed. However, disease progression, predominantly at distant sites, remained common. An HPV-positive oropharyngeal primary tumor was associated with a favorable prognosis, whereas metastases at non-lung sites and a higher metastatic burden were adverse factors. Additionally, host nutritional and inflammatory indices, including their rate of change, were also associated with outcomes. We propose an exploratory PMFS nomogram based on four routinely available clinical variables that may inform patient selection for aggressive local therapy; this tool requires external validation before clinical application.

## Figures and Tables

**Table 1:** Baseline characteristics of the entire cohort.

Characteristic	Total (N = 93)
Age, years—median [IQR]	59 [55–66]
**Sex**	
Male	76 (81.7%)
Female	17 (18.3%)
**Smoking**	
Never	33 (35.5%)
Former	32 (34.4%)
Current	28 (30.1%)
**Alcohol**	
Yes	51 (54.8%)
No/former	42 (45.2%)
**Primary group**	
Nasopharynx	27 (29.0%)
HPV-positive oropharynx	11 (11.8%)
Others	55 (59.1%)
**Primary site**	
Nasopharynx	27 (29.0%)
Oropharynx	21 (22.6%)
Larynx	15 (16.1%)
Oral cavity	14 (15.1%)
Hypopharynx	11 (11.8%)
Sinonasal	4 (4.3%)
Oral cavity + nasopharynx	1 (1.1%)
**p16/HPV status in oropharyngeal carcinoma (*n* = 21)**	
Positive	11 (52.4%)
Negative	6 (28.6%)
Unknown	4 (19.0%)
**EBV status in nasopharyngeal carcinoma (*n* = 27)**	
Positive	20 (74.1%)
Negative	1 (3.7%)
Unknown	6 (22.2%)
**Disease scenario**	
Metachronous	81 (87.1%)
Synchronous	12 (12.9%)
Disease-free interval, mo (metachronous, *n* = 81)	21.3 [13.1–34.0]
**No. of metastases**	
1	77 (82.8%)
≥2	16 (17.2%)
**No. of involved organs**	
1	91 (97.8%)
2	2 (2.2%)
Largest metastasis, cm	1.6 [1.2–2.5]
**Metastatic site**	
Lung only	65 (69.9%)
Non-lung	28 (30.1%)
Bone	12 (12.9%)
Liver	6 (6.5%)
Distant lymph node	5 (5.4%)
Other	3 (3.2%)
Multiple sites	2 (2.2%)
**Local therapy modality**	
SBRT	41 (44.1%)
Surgery	34 (36.6%)
Conventional RT	13 (14.0%)
RFA	5 (5.4%)
**Treatment intent**	
Curative/upfront	86 (92.5%)
Salvage/other	7 (7.5%)
BED10, Gy (RT subset, *n* = 51)	112.5 [75.0–150.0]
**Systemic therapy**	
Pre-local	20 (21.5%)
Concurrent	9 (9.7%)
Post-local	7 (7.5%)
**Sarcopenia @OM**	
Cohort cutoff	30 (32.3%)
Troschel cutoff	72 (77.4%)
**Treatment era**	
≤2015	32 (34.4%)
2016–2020	27 (29.0%)
2021–2025	34 (36.6%)

Continuous variables are median [interquartile range]; categorical variables are *n* (%). @OM, at oligometastasis diagnosis; SBRT, stereotactic body radiotherapy; RT, radiotherapy; RFA, radiofrequency ablation; BED, biologically effective dose; HPV, human papillomavirus; EBV, Epstein–Barr virus. Percentages for p16/HPV and EBV status were calculated within the oropharyngeal and nasopharyngeal carcinoma subgroups, respectively.

**Table 2:** Patterns of failure.

	*n* (%)
Any disease progression	64 (68.8)
**First site of failure**	
Distant metastasis, alone	51 (54.8)
Head and neck recurrence, alone	5 (5.4)
Local failure, alone	4 (4.3)
Combined sites	4 (4.3)
**Sites of failure (overall)**	
Local failure (treated metastatic lesion)	9 (9.7)
Head and neck recurrence	8 (8.6)
Distant metastasis, same organ	28 (30.1)
Distant metastasis, different organ	14 (15.1)
Distant metastasis, both	15 (16.1)

Data are *n* (%) of the whole cohort. Organ concordance is relative to the metastatic site(s) involved at oligometastatic presentation.

**Table 3:** Univariable and multivariable analysis of prognostic factors in the entire cohort.

	Univariable		Multivariable	
	HR (95% CI)	*p* Value	HR (95% CI)	*p* Value
**Overall survival**				
Age (per year)	1.02 (0.99–1.04)	0.184	–	–
Female	1.10 (0.57–2.11)	0.783	–	–
Charlson index (per point)	1.28 (0.86–1.89)	0.223	–	–
Nasopharynx (vs. others)	0.76 (0.41–1.38)	0.362	0.76 (0.41–1.38)	0.362
HPV-positive oropharynx (vs. others)	**0.23 (0.05–0.95)**	**0.042**	**0.23 (0.05–0.95)**	**0.042**
Synchronous (vs. metachronous)	1.11 (0.52–2.36)	0.796	–	–
DFI (per month)	1.01 (1.00–1.02)	0.162	–	–
Metastases ≥ 2 (vs. 1)	1.56 (0.75–3.24)	0.231	–	–
Largest metastasis size (per cm)	1.15 (0.96–1.38)	0.139	–	–
Non-lung site (vs. lung-only)	1.45 (0.82–2.57)	0.206	–	–
Residual head and neck disease (vs. NED)	1.07 (0.53–2.15)	0.847	–	–
SMI@OM (per unit)	1.00 (0.97–1.03)	0.912	–	–
Albumin@OM (per g/dL)	0.56 (0.23–1.37)	0.205	–	–
Hemoglobin@OM (per g/dL)	0.89 (0.74–1.09)	0.264	–	–
PNI@OM (per point)	0.99 (0.92–1.05)	0.679	–	–
**Progression-free survival**				
Age (per year)	1.01 (0.99–1.03)	0.365	–	–
Female	1.32 (0.73–2.38)	0.354	–	–
Charlson index (per point)	1.20 (0.90–1.61)	0.204	–	–
Nasopharynx (vs. others)	0.97 (0.57–1.64)	0.909	–	–
HPV-positive oropharynx (vs. others)	0.42 (0.17–1.06)	0.067	–	–
Synchronous (vs. metachronous)	1.84 (0.95–3.56)	0.069	–	–
DFI (per month)	1.00 (0.99–1.01)	0.870	–	–
Metastases ≥ 2 (vs. 1)	1.30 (0.70–2.43)	0.412	–	–
Largest metastasis size (per cm)	1.08 (0.92–1.27)	0.355	–	–
Non-lung site (vs. lung-only)	1.54 (0.94–2.53)	0.086	**1.78 (1.06–2.99)**	**0.030**
Residual head and neck disease (vs. NED)	1.70 (0.92–3.14)	0.091	–	–
SMI@OM (per unit)	0.99 (0.96–1.02)	0.481	–	–
Albumin@OM (per g/dL)	**0.32 (0.14–0.73)**	**0.007**	**0.42 (0.18–0.98)**	**0.046**
Hemoglobin@OM (per g/dL)	0.85 (0.72–1.01)	0.063	0.87 (0.73–1.05)	0.152
PNI@OM (per point)	0.96 (0.90–1.01)	0.142	–	–
**Polymetastasis-free survival**				
Age (per year)	1.01 (0.99–1.03)	0.557	–	–
Female	1.38 (0.72–2.64)	0.327	–	–
Charlson index (per point)	1.19 (0.84–1.70)	0.327	–	–
Nasopharynx (vs. others)	1.19 (0.67–2.10)	0.547	0.86 (0.44–1.67)	0.651
HPV-positive oropharynx (vs. others)	**0.22 (0.05–0.93)**	**0.039**	**0.22 (0.05–0.93)**	**0.039**
Synchronous (vs. metachronous)	1.23 (0.58–2.64)	0.587	–	–
DFI (per month)	1.01 (1.00–1.02)	0.218	–	–
Metastases ≥ 2 (vs. 1)	1.76 (0.88–3.52)	0.110	–	–
Largest metastasis size (per cm)	**1.19 (1.00–1.41)**	**0.046**	–	–
Non-lung site (vs. lung-only)	**1.92 (1.12–3.30)**	**0.019**	1.88 (1.00–3.55)	0.051
Residual head and neck disease (vs. NED)	1.20 (0.60–2.40)	0.610	–	–
SMI@OM (per unit)	1.00 (0.97–1.03)	0.769	–	–
Albumin@OM (per g/dL)	0.61 (0.25–1.46)	0.265	–	–
Hemoglobin@OM (per g/dL)	0.89 (0.74–1.07)	0.205	–	–
PNI@OM (per point)	0.98 (0.92–1.05)	0.596	–	–

Because of missing covariate values, the multivariable model for progression-free survival was fitted on 89 cases. Selected univariable results are shown; all variables examined are presented in Supplementary Table S3, from which multivariable candidates (univariable *p* < 0.10) were drawn. Bold, *p* < 0.05. HR, hazard ratio; DFI, disease-free interval; NED, no evidence of disease; SMI, skeletal muscle index; PNI, prognostic nutritional index.

**Table 4:** Univariable and multivariable analysis of prognostic factors in the metachronous subset (*n* = 81).

	Univariable		Multivariable	
	HR (95% CI)	*p* Value	HR (95% CI)	*p* Value
**Overall survival**				
Age (per year)	1.02 (0.99–1.04)	0.162	–	–
Female	1.28 (0.63–2.62)	0.499	–	–
Charlson index (per point)	1.28 (0.83–1.97)	0.258	–	–
Nasopharynx (vs. others)	0.66 (0.31–1.40)	0.283	0.46 (0.20–1.02)	0.057
HPV-positive oropharynx (vs. others)	**0.23 (0.05–0.94)**	**0.041**	**0.23 (0.05–0.96)**	**0.044**
DFI (per month)	1.01 (1.00–1.02)	0.105	–	–
Metastases ≥ 2 (vs. 1)	1.58 (0.75–3.34)	0.228	–	–
Largest metastasis size (per cm)	1.16 (0.95–1.41)	0.147	–	–
Non-lung site (vs. lung-only)	1.61 (0.82–3.16)	0.163	–	–
Residual head and neck disease (vs. NED)	1.20 (0.37–3.91)	0.767	–	–
SMI@OM (per unit)	1.00 (0.96–1.03)	0.866	–	–
Albumin@OM (per g/dL)	0.39 (0.14–1.07)	0.069	**0.36 (0.14–0.96)**	**0.042**
Hemoglobin@OM (per g/dL)	0.87 (0.71–1.08)	0.205	–	–
PNI@OM (per point)	0.95 (0.89–1.03)	0.210	–	–
NLR rate (per SD)	**1.34 (1.01–1.77)**	**0.044**	–	–
SII rate (per SD)	**1.43 (1.04–1.96)**	**0.030**	1.32 (0.96–1.81)	0.089
**Progression-free survival**				
Age (per year)	1.01 (0.99–1.04)	0.190	–	–
Female	1.30 (0.69–2.46)	0.418	–	–
Charlson index (per point)	1.25 (0.93–1.70)	0.144	–	–
Nasopharynx (vs. others)	0.77 (0.41–1.44)	0.414	–	–
HPV-positive oropharynx (vs. others)	0.43 (0.17–1.08)	0.072	–	–
DFI (per month)	1.00 (0.99–1.01)	0.618	–	–
Metastases ≥ 2 (vs. 1)	1.42 (0.75–2.69)	0.285	–	–
Largest metastasis size (per cm)	1.09 (0.92–1.29)	0.329	–	–
Non-lung site (vs. lung-only)	1.34 (0.76–2.39)	0.315	–	–
Residual head and neck disease (vs. NED)	1.56 (0.56–4.34)	0.393	–	–
SMI@OM (per unit)	0.99 (0.96–1.02)	0.423	–	–
Albumin@OM (per g/dL)	**0.25 (0.10–0.64)**	**0.004**	**0.25 (0.10–0.64)**	**0.004**
Hemoglobin@OM (per g/dL)	**0.82 (0.68–0.99)**	**0.037**	–	–
PNI@OM (per point)	**0.93 (0.88–0.99)**	**0.033**	–	–
NLR rate (per SD)	**1.35 (1.04–1.76)**	**0.026**	–	–
SII rate (per SD)	1.24 (0.90–1.71)	0.184	–	–
**Polymetastasis-free survival**				
Age (per year)	1.01 (0.99–1.04)	0.333	–	–
Female	1.35 (0.67–2.74)	0.398	–	–
Charlson index (per point)	1.21 (0.83–1.77)	0.324	–	–
Nasopharynx (vs. others)	1.09 (0.56–2.12)	0.806	0.66 (0.31–1.41)	0.287
HPV-positive oropharynx (vs. others)	**0.22 (0.05–0.92)**	**0.039**	0.26 (0.06–1.12)	0.071
DFI (per month)	1.01 (1.00–1.02)	0.102	–	–
Metastases ≥ 2 (vs. 1)	1.80 (0.89–3.67)	0.104	–	–
Largest metastasis size (per cm)	**1.21 (1.01–1.44)**	**0.039**	–	–
Non-lung site (vs. lung-only)	**1.97 (1.06–3.67)**	**0.032**	1.72 (0.87–3.41)	0.118
Residual head and neck disease (vs. NED)	1.06 (0.33–3.45)	0.919	–	–
SMI@OM (per unit)	0.99 (0.96–1.03)	0.716	–	–
Albumin@OM (per g/dL)	0.48 (0.18–1.28)	0.142	–	–
Hemoglobin@OM (per g/dL)	0.86 (0.71–1.05)	0.137	–	–
PNI@OM (per point)	0.97 (0.90–1.03)	0.323	–	–
NLR rate (per SD)	**1.28 (1.01–1.63)**	**0.043**	–	–
SII rate (per SD)	**1.34 (1.02–1.77)**	**0.039**	1.33 (0.98–1.81)	0.067

Because of missing covariate values, the multivariable models for the affected endpoints were fitted on 71 cases for overall survival and polymetastasis-free survival, and 78 for progression-free survival. Selected univariable results are shown; all variables examined are presented in Supplementary Table S5, from which multivariable candidates (univariable *p* < 0.10) were drawn. Bold, *p* < 0.05. HR, hazard ratio; DFI, disease-free interval; NED, no evidence of disease; SMI, skeletal muscle index; PNI, prognostic nutritional index; NLR, neutrophil-to-lymphocyte ratio; SII, systemic immune-inflammation index.

## Data Availability

The data presented in this study are available on request from the corresponding author due to privacy and ethical restrictions.

## Supplementary Materials

The following supporting information can be downloaded at: https://www.mdpi.com/article/10.3390/curroncol33100585/s1, Table S1: Dose/fractionation schedules for stereotactic body radiotherapy and conventional radiotherapy; Table S2: Body composition indices and inflammatory/nutritional markers at diagnosis and the time of oligometastasis diagnosis; Table S3: Univariable analysis of prognostic factors in the entire cohort (N = 93); Table S4: Fine-Gray subdistribution hazard analysis for polymetastatic conversion; Table S5: Univariable analysis of prognostic factors in the metachronous subset (*n* = 81); Figure S1: Kaplan–Meier curves of overall survival (left), progression-free survival (middle), and polymetastasis-free survival (right) according to primary tumor group: HPV-positive oropharynx (*n* = 11), nasopharynx (*n* = 27), and other primaries (*n* = 55). *p* values were calculated using the log-rank test. Numbers at risk are shown below each panel.
