# Supplementary material for: Prognostic Factors and a Polymetastasis-Free Survival Nomogram in Oligometastatic Head and Neck Squamous Cell Carcinoma Treated with Local Ablative Therapy
Source: Curr Oncol. 2026 Sep 29;33(10):585. doi: 10.3390/curroncol33100585 (PMC13703399; doi:10.3390/curroncol33100585)
Supplement: Supplementary file 1 [file curroncol-33-00585-s001.zip › curroncol-4512938-supplementary.pdf]

Supplementary Table S1. Dose/fractionation schedules for stereotactic body radiotherapy and conventional radiotherapy.

Treatment modality	Total dose / number of fractions	Patients, n
SBRT	32 Gy / 4 fractions	1
	32.5 Gy / 5 fractions	1
	33 Gy / 3 fractions	2
	42 Gy / 3 fractions	1
	45 Gy / 3 fractions	1
	48 Gy / 4 fractions	4
	50 Gy / 10 fractions	3
	52 Gy / 4 fractions	2
	52 Gy / 4 fractions and 60 Gy / 4 fractions	1
	56 Gy / 4 fractions	4
	60 Gy / 4 fractions	21
**SBRT subtotal**		**41**
Conventional radiotherapy	33 Gy / 10 fractions	1
	36 Gy / 12 fractions	2
	39 Gy / 13 fractions	1
	45 Gy / 15 fractions	1
	60 Gy / 30 fractions	1
	63.8 Gy / 29 fractions	1
	64 Gy / 32 fractions	1
	66 Gy / 30 fractions	1
	66 Gy / 33 fractions	1
	70 Gy / 35 fractions	2
	70.7 Gy / 34 fractions	1
**Conventional radiotherapy subtotal**		**13**
**Total**		**54**

Abbreviation: SBRT, stereotactic body radiotherapy. Counts represent patients. A patient with two recorded schedules is counted once in the combined row.

Supplementary Table S2. Body composition indices and inflammatory/nutritional markers at diagnosis and the time of oligometastasis diagnosis.

Marker	At diagnosis	At oligometastasis	Δ (absolute)	Rate/month	n
T12 SMI, cm²/m²	37.3 [31.0–41.5]	34.3 [29.3–39.5]	−1.9 [−4.2–0.0]	−0.09 [−0.22–0.02]	93 / 75
T12 CSA, cm²	102.5 [82.7–115.6]	94.1 [77.7–110.8]	−4.8 [−11.1–0.0]	−0.26 [−0.60–0.05]	93 / 75
NLR	2.06 [1.60–3.00]	2.41 [1.74–3.55]	+0.27 [−0.36–1.20]	+0.02 [−0.03–0.06]	89 / 71
PNI	50.4 [46.1–54.0]	46.7 [43.8–50.6]	−2.3 [−5.9–0.0]	−0.11 [−0.26–−0.02]	89 / 71
SII	492 [352–815]	503 [357–732]	0 [−188–175]	+1.33 [−11.6–8.0]	89 / 71
Albumin, g/dL	4.0 [3.8–4.3]	4.0 [3.7–4.2]	0.0 [−0.3–0.1]	−0.00 [−0.01–0.01]	90 / 71
Hemoglobin, g/dL	13.5 [12.8–14.6]	13.0 [12.0–14.2]	−0.3 [−1.1–0.2]	−0.02 [−0.06–0.01]	89 / 71
Platelets, ×10³/μL	248 [204–279]	215 [171–250]	−32 [−59–0]	−1.8 [−3.5–−0.6]	89 / 71

Values are median [interquartile range]. Δ, change from diagnosis to oligometastasis; rate, Δ per month among the 81 metachronous patients. n, available at oligometastasis / rate estimable. SMI, skeletal muscle index; CSA, cross-sectional area; NLR, neutrophil-to-lymphocyte ratio; PNI, prognostic nutritional index; SII, systemic immune-inflammation index.

Supplementary Table S3. Univariable analysis of prognostic factors in the entire cohort (N = 93).

Univariable Cox proportional-hazards models for overall survival (OS), progression-free survival (PFS), and polymetastasis-free survival (PMFS). Values at oligometastasis (@OM) are per native unit; rate-of-change indices are per 1 standard deviation. Bold, p < 0.05. Calendar-period and treatment variables were not analyzed; polymetastatic conversion is shown in Supplementary Table S4. HR, hazard ratio; CI, confidence interval; OMD, oligometastasis; DFI, disease-free interval; NED, no evidence of disease; SMI, skeletal muscle index; NLR, neutrophil-to-lymphocyte ratio; SII, systemic immune-inflammation index; PNI, prognostic nutritional index.

**Variable**	**OS**		**PFS**		**PMFS**	
	**HR (95% CI)**	**p**	**HR (95% CI)**	**p**	**HR (95% CI)**	**p**
Demographics/comorbidity						
Age (per year)	1.02 (0.99–1.04)	0.184	1.01 (0.99–1.03)	0.365	1.01 (0.99–1.03)	0.557
Female	1.10 (0.57–2.11)	0.783	1.32 (0.73–2.38)	0.354	1.38 (0.72–2.64)	0.327
Ever-smoker	1.00 (0.56–1.76)	0.988	0.80 (0.49–1.30)	0.362	0.87 (0.50–1.51)	0.620
Charlson index (per point)	1.28 (0.86–1.89)	0.223	1.20 (0.90–1.61)	0.204	1.19 (0.84–1.70)	0.327
Tumor/disease						
Nasopharynx (vs others)	0.76 (0.41–1.38)	0.362	0.97 (0.57–1.64)	0.909	1.19 (0.67–2.10)	0.547
HPV+ oropharynx (vs others)	**0.23 (0.05–0.95)**	**0.042**	0.42 (0.17–1.06)	0.067	**0.22 (0.05–0.93)**	**0.039**
Synchronous	1.11 (0.52–2.36)	0.796	1.84 (0.95–3.56)	0.069	1.23 (0.58–2.64)	0.587
DFI (per month)	1.01 (1.00–1.02)	0.162	1.00 (0.99–1.01)	0.870	1.01 (1.00–1.02)	0.218
Metastases ≥2	1.56 (0.75–3.24)	0.231	1.30 (0.70–2.43)	0.412	1.76 (0.88–3.52)	0.110
Largest size (per cm)	1.15 (0.96–1.38)	0.139	1.08 (0.92–1.27)	0.355	**1.19 (1.00–1.41)**	**0.046**
Non-lung site	1.45 (0.82–2.57)	0.206	1.54 (0.94–2.53)	0.086	**1.92 (1.12–3.30)**	**0.019**
Residual head and neck disease (vs NED)	1.07 (0.53–2.15)	0.847	1.70 (0.92–3.14)	0.091	1.20 (0.60–2.40)	0.610
Body composition/nutrition/inflammation						
Sarcopenia	1.01 (0.56–1.81)	0.979	1.02 (0.61–1.68)	0.951	1.12 (0.64–1.96)	0.687
SMI@OM (per unit)	1.00 (0.97–1.03)	0.912	0.99 (0.96–1.02)	0.481	1.00 (0.97–1.03)	0.769
NLR@OM (per unit)	1.01 (0.84–1.22)	0.908	1.07 (0.93–1.23)	0.352	1.03 (0.87–1.21)	0.753
SII@OM (per unit)	1.00 (1.00–1.00)	0.717	1.00 (1.00–1.00)	0.314	1.00 (1.00–1.00)	0.698
Albumin@OM (per g/dL)	0.56 (0.23–1.37)	0.205	**0.32 (0.14–0.73)**	**0.007**	0.61 (0.25–1.46)	0.265
Hemoglobin@OM (per g/dL)	0.89 (0.74–1.09)	0.264	0.85 (0.72–1.01)	0.063	0.89 (0.74–1.07)	0.205
PNI@OM (per point)	0.99 (0.92–1.05)	0.679	0.96 (0.90–1.01)	0.142	0.98 (0.92–1.05)	0.596

Supplementary Table S4. Fine-Gray subdistribution hazard analysis for polymetastatic conversion.

	Univariable		Multivariable	
	**sHR (95% CI)**	**P value**	**sHR (95% CI)**	**P value**
**Whole cohort (N = 93)**				
Age (per year)	**0.98 (0.96–1.00)**	**0.043**	–	–
Female	**2.01 (1.05–3.85)**	**0.036**	1.81 (0.90–3.65)	0.098
Charlson index (per point)	0.97 (0.62–1.54)	0.911	–	–
Nasopharynx (vs others)	**1.86 (1.01–3.41)**	**0.046**	0.93 (0.40–2.14)	0.858
HPV-positive oropharynx (vs others)	0.21 (0.03–1.54)	0.125	0.19 (0.02–1.49)	0.114
Synchronous (vs metachronous)	1.17 (0.55–2.45)	0.686	–	–
DFI (per month)	1.01 (1.00–1.01)	0.107	–	–
Metastases ≥2 (vs 1)	**2.87 (1.32–6.24)**	**0.008**	**2.51 (1.22–5.16)**	**0.012**
Largest metastasis size (per cm)	**1.25 (1.03–1.53)**	**0.023**	–	–
Non-lung site (vs lung-only)	**2.48 (1.36–4.53)**	**0.003**	**2.25 (1.09–4.65)**	**0.029**
Residual head and neck disease (vs NED)	1.28 (0.64–2.54)	0.486	–	–
SMI@OM (per unit)	1.11 (0.89–1.38)	0.338	–	–
Albumin@OM (per g/dL)	1.04 (0.75–1.43)	0.828	–	–
Hemoglobin@OM (per g/dL)	1.01 (0.75–1.34)	0.972	–	–
PNI@OM (per point)	1.09 (0.80–1.48)	0.585	–	–
**Metachronous subset (n = 81)**				
Age (per year)	0.98 (0.96–1.00)	0.123	–	–
Female	1.91 (0.92–3.99)	0.084	–	–
Charlson index (per point)	0.91 (0.51–1.62)	0.739	–	–
Nasopharynx (vs others)	1.70 (0.86–3.37)	0.129	–	–
HPV-positive oropharynx (vs others)	0.20 (0.03–1.42)	0.107	–	–
DFI (per month)	**1.01 (1.00–1.01)**	**0.017**	**1.01 (1.00–1.02)**	**0.003**
Metastases ≥2 (vs 1)	**2.97 (1.36–6.45)**	**0.006**	**3.59 (1.72–7.49)**	**<0.001**
Largest metastasis size (per cm)	**1.29 (1.05–1.58)**	**0.017**	**1.32 (1.10–1.58)**	**0.003**
Non-lung site (vs lung-only)	**2.42 (1.21–4.81)**	**0.012**	–	–
Residual head and neck disease (vs NED)	0.89 (0.23–3.46)	0.862	–	–
SMI@OM (per unit)	1.11 (0.87–1.43)	0.389	–	–
Albumin@OM (per g/dL)	0.96 (0.68–1.35)	0.799	–	–
Hemoglobin@OM (per g/dL)	0.92 (0.68–1.25)	0.613	–	–
PNI@OM (per point)	1.03 (0.75–1.40)	0.868	–	–
NLR rate (per SD)	1.12 (0.84–1.49)	0.425	–	–
SII rate (per SD)	1.20 (0.89–1.63)	0.228	–	–

Bold, $p < 0.05$. sHR, subdistribution hazard ratio; OMD, oligometastasis; DFI, disease-free interval; NED, no evidence of disease; SMI, skeletal muscle index; PNI, prognostic nutritional index; NLR, neutrophil-to-lymphocyte ratio; SII, systemic immune-inflammation index.

Supplementary Table S5. Univariable analysis of prognostic factors in the metachronous subset (n = 81).

Univariable Cox proportional-hazards models in the metachronous subset for overall survival (OS), progression-free survival (PFS), and polymetastasis-free survival (PMFS). Values at oligometastasis (@OM) are per native unit; rate-of-change indices are per 1 standard deviation of the change from diagnosis to oligometastasis. Bold, p < 0.05. Calendar-period and treatment variables were not analyzed; polymetastatic conversion is shown in Supplementary Table S4. HR, hazard ratio; CI, confidence interval; OMD, oligometastasis; DFI, disease-free interval; NED, no evidence of disease; SMI, skeletal muscle index; NLR, neutrophil-to-lymphocyte ratio; SII, systemic immune-inflammation index; PNI, prognostic nutritional index.

Variable	OS		PFS		PMFS	
	HR (95% CI)	p	HR (95% CI)	p	HR (95% CI)	p
Demographics/comorbidity						
Age (per year)	1.02 (0.99–1.04)	0.162	1.01 (0.99–1.04)	0.190	1.01 (0.99–1.04)	0.333
Female	1.28 (0.63–2.62)	0.499	1.30 (0.69–2.46)	0.418	1.35 (0.67–2.74)	0.398
Ever-smoker	0.95 (0.50–1.82)	0.886	0.87 (0.50–1.51)	0.631	0.91 (0.49–1.70)	0.776
Charlson index (per point)	1.28 (0.83–1.97)	0.258	1.25 (0.93–1.70)	0.144	1.21 (0.83–1.77)	0.324
Tumor/disease						
Nasopharynx (vs others)	0.66 (0.31–1.40)	0.283	0.77 (0.41–1.44)	0.414	1.09 (0.56–2.12)	0.806
HPV+ oropharynx (vs others)	**0.23 (0.05–0.94)**	**0.041**	0.43 (0.17–1.08)	0.072	**0.22 (0.05–0.92)**	**0.039**
DFI (per month)	1.01 (1.00–1.02)	0.105	1.00 (0.99–1.01)	0.618	1.01 (1.00–1.02)	0.102
Metastases ≥2	1.58 (0.75–3.34)	0.228	1.42 (0.75–2.69)	0.285	1.80 (0.89–3.67)	0.104
Largest size (per cm)	1.16 (0.95–1.41)	0.147	1.09 (0.92–1.29)	0.329	**1.21 (1.01–1.44)**	**0.039**
Non-lung site	1.61 (0.82–3.16)	0.163	1.34 (0.76–2.39)	0.315	**1.97 (1.06–3.67)**	**0.032**
Residual head and neck disease (vs NED)	1.20 (0.37–3.91)	0.767	1.56 (0.56–4.34)	0.393	1.06 (0.33–3.45)	0.919
Body composition/nutrition/inflammation						
Sarcopenia	1.09 (0.58–2.05)	0.783	1.03 (0.60–1.77)	0.924	1.15 (0.63–2.09)	0.640
SMI@OM (per unit)	1.00 (0.96–1.03)	0.866	0.99 (0.96–1.02)	0.423	0.99 (0.96–1.03)	0.716
NLR@OM (per unit)	1.09 (0.90–1.32)	0.394	1.07 (0.93–1.24)	0.338	1.06 (0.89–1.25)	0.528
SII@OM (per unit)	1.00 (1.00–1.00)	0.668	1.00 (1.00–1.00)	0.419	1.00 (1.00–1.00)	0.939
Albumin@OM (per g/dL)	0.39 (0.14–1.07)	0.069	**0.25 (0.10–0.64)**	**0.004**	0.48 (0.18–1.28)	0.142
Hemoglobin@OM (per g/dL)	0.87 (0.71–1.08)	0.205	**0.82 (0.68–0.99)**	**0.037**	0.86 (0.71–1.05)	0.137
PNI@OM (per point)	0.95 (0.89–1.03)	0.210	**0.93 (0.88–0.99)**	**0.033**	0.97 (0.90–1.03)	0.323
Rate of change (per month, per SD)						
NLR rate (per SD)	**1.34 (1.01–1.77)**	**0.044**	**1.35 (1.04–1.76)**	**0.026**	**1.28 (1.01–1.63)**	**0.043**
SII rate (per SD)	**1.43 (1.04–1.96)**	**0.030**	1.24 (0.90–1.71)	0.184	**1.34 (1.02–1.77)**	**0.039**
SMI rate (per SD)	1.17 (0.78–1.75)	0.451	1.05 (0.78–1.42)	0.740	1.10 (0.79–1.54)	0.577
CSA rate (per SD)	1.14 (0.77–1.69)	0.517	1.05 (0.78–1.41)	0.746	1.08 (0.77–1.51)	0.648
Albumin rate (per SD)	0.94 (0.63–1.40)	0.757	1.00 (0.73–1.38)	0.995	0.99 (0.70–1.42)	0.969
Hemoglobin rate (per SD)	1.46 (0.84–2.55)	0.182	1.45 (0.90–2.34)	0.123	1.46 (0.85–2.52)	0.174
PNI rate (per SD)	0.85 (0.58–1.26)	0.423	0.94 (0.68–1.28)	0.685	0.88 (0.61–1.25)	0.472
PLT rate (per SD)	1.30 (0.80–2.11)	0.292	0.92 (0.68–1.23)	0.560	1.18 (0.78–1.77)	0.442

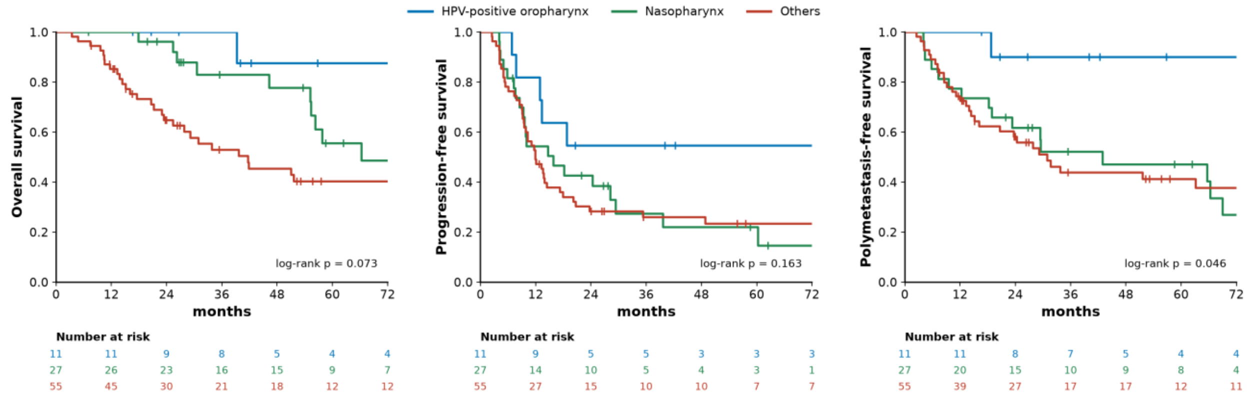

Supplementary Figure S1. Kaplan–Meier curves of overall survival (left), progression-free survival (middle), and polymetastasis-free survival (right) according to primary tumor group: HPV-positive oropharynx (n = 11), nasopharynx (n = 27), and other primaries (n = 55). P values were calculated using the log-rank test. Numbers at risk are shown below each panel.
